# In vivo label-free optical monitoring of structural and metabolic remodeling of myocardium following infarction

**DOI:** 10.1364/BOE.10.003506

**Published:** 2019-06-21

**Authors:** João L. Lagarto, Benjamin T. Dyer, Nicholas S. Peters, Paul M. W. French, Chris Dunsby, Alexander R. Lyon

**Affiliations:** 1 Photonics Group, Department of Physics, Imperial College London, Prince Consort Road, London, SW7 2AZ, United Kingdom; 2 National Heart and Lung Institute, Imperial College London, Du Cane Road, London, W12 0NN, United Kingdom; 3 Centre for Cardiac Engineering, Imperial College London, Du Cane Road, London, W12 0NN, United Kingdom; 4 Centre for Pathology, Imperial College London Du Cane Road, London W12 0NN, United Kingdom; 5 Authors contributed equally to this work.; 6 Authors contributed equally to this work.

## Abstract

Cardiac remodeling following myocardial infarction (MI) involves structural and functional alterations in the infarcted and remote viable myocardium that can ultimately lead to heart failure. The underlying mechanisms are not fully understood and, following our previous study of the autofluorescence lifetime and diffuse reflectance signatures of the myocardium in vivo at 16 weeks post MI in rats [Biomed. Opt. Express 6(2), 324 (2015)2578072710.1364/BOE.6.000324PMC4354591], we here present data obtained at 1, 2 and 4 weeks post myocardial infarction that help follow the temporal progression of these changes. Our results demonstrate that both structural and metabolic changes in the heart can be monitored from the earliest time points following MI using label-free optical readouts, not only in the region of infarction but also in the remote non-infarcted myocardium. Changes in the autofluorescence intensity and lifetime parameters associated with collagen type I autofluorescence were indicative of progressive collagen deposition in tissue that was most pronounced at earlier time points and in the region of infarction. In addition to significant collagen deposition in infarcted and non-infarcted myocardium, we also report changes in the autofluorescence parameters associated with reduced nicotinamide adenine (phosphate) dinucleotide (NAD(P)H) and flavin adenine dinucleotide (FAD), which we associate with metabolic alterations throughout the heart. Parallel measurements of the diffuse reflectance spectra indicated an increased contribution of reduced cytochrome c. Our findings suggest that combining time-resolved spectrofluorometry and diffuse reflectance spectroscopy could provide a useful means to monitor cardiac function in vivo at the time of surgery.

## 1. Introduction

Myocardial infarction (MI) following coronary artery disease remains the most common cause of cardiovascular disease and is responsible for a high rate of hospitalization and mortality, making it a leading cause of death worldwide [1]. Following MI, a number of repair mechanisms try to maintain the structural integrity and viability of the infarcted myocardium, including replacement of injured tissue by a fibrotic scar and remodeling of the remaining ventricle [2–4]. This ventricular remodeling can change the shape, size, structure of the heart and decrease the contractile performance, leading to heart failure [5]. While the mechanisms of cardiac remodeling are not completely understood, they involve complex and dynamic functional, morphological and biochemical alterations [6,7]. These are not restricted to the region of infarction but can also affect the remote non-infarcted myocardium, initially compensating for increased wall stress but ultimately driving the progression from compensatory hypertrophy to heart failure.

In recent years, myocardial structural and functional abnormalities have been extensively characterized. Reported changes include alterations of interstitial fibrosis throughout the heart affecting tissue compliance, and contraction and relaxation [4], alteration of the electrical properties of the tissue [8] as well as alteration of the mitochondrial function and energy production of the failing heart [9]. After an acute myocardial injury such as myocardial infarction of the left ventricle, the heart undergoes structural and metabolic remodeling to compensate for the loss of function in the infarcted area. The wound-healing process is initiated within a few hours after the injury occurs and localized to the area of infarction [10,11]. The initial remodeling phase consists of the repair of the lesion via deposition of collagen types III and I and rearrangement of the collagenous matrix, i.e. scar formation, which is promoted by the proliferation and upregulation of myofibroblasts [12]. Deposition of collagen types III and I has been observed from just two and four days post-infarction, respectively, and can still be observed after three months, in the case of collagen I, depending on the severity of the lesion [3,10–12]. The deposition of collagen increases the tensile strength of the muscle allowing maintenance of the cardiac output with concomitant increase in energy consumption. Progressive loss of contractile function due to increased stiffness further increases the energy consumption as the heart tries to maintain the cardiac output. Eventually, the energy produced is not sufficient to meet the demand, resulting in dysregulation of cellular processes and further loss of function, ultimately leading to heart failure. While hallmark structural and functional abnormalities of the myocardium can be evaluated using currently available clinical tools (e.g. fluorodeoxyglucose positron emission tomography (FDG-PET) [13], single photon emission computed tomography (SPECT) [14], gadolinium enhancement cardiac magnetic resonance imaging (LGE-CMRI) [15]), these technologies typically involve bulky and expensive instrumentation that is not suitable for in situ and real time characterization of myocardial structure and function in surgical settings.

Label-free optical spectroscopy is an attractive approach to study biological tissues due to its potential low-cost and small size compared to conventional clinical imaging modalities. Previous research in this area has exploited diffuse reflectance spectroscopy to probe dynamics of the myocardium e.g [16–18]. or steady-state autofluorescence spectroscopy to monitor tissue viability following MI [19,20]. Autofluorescence lifetime spectroscopy offers further opportunities to monitor myocardial viability, since the fluorescence decay characteristics of endogenous fluorophores (e.g. reduced nicotinamide adenine (phosphate) dinucleotide (NAD(P)H), flavin adenine dinucleotide (FAD), tryptophan, collagens, elastin, porphyrins) are highly sensitive to structural and biochemical properties of the tissue. Since they are inherently ratiometric, i.e. they are derived from the relative signal level at different times following excitation, autofluorescence lifetime measurements are relatively insensitive to the artefacts that can compromise absolute intensity measurements such as variable excitation-collection geometry, fluorophore concentration or heterogenous optical absorption [21]. Furthermore, it can improve the specificity of autofluorescence measurements and provide means to distinguish fluorophores that have broadly overlapping fluorescence emission spectra but different decay times. Accordingly, autofluorescence lifetime spectroscopy has demonstrated clinical potential in a number of applications both ex vivo (e.g [22,23]) and in vivo (e.g [24–27]), by providing label-free structural and functional information of biological tissues. The fluorescence lifetime of endogenous fluorescence can be measured via single point measurements (e.g [24,28]), or can be extended to fluorescence lifetime imaging (e.g [22,27,29]).

In a previous study, we exploited the potential of a multimodal setup combining autofluorescence lifetime and diffuse reflectance spectroscopy to report structural and functional alterations in the myocardium in a 16-week myocardial infarction heart failure model in rats [30]. While we were able to identify structural changes in the heart that we attributed to increases in collagen, both in the infarcted area and in the non-infarcted remote myocardium, it was significantly more difficult to discern changes in the energetic state. The autofluorescence signal emanating from healthy cardiac tissue is dominated by NAD(P)H and FAD, while extracellular matrix components (collagens type I and III and elastin) play a less relevant role. At 16 weeks post-infarction the contribution from NAD(P)H and FAD to the autofluorescence signal decreases significantly as a result of extensive cardiomyocyte loss and collagen proliferation, making it challenging to quantify changes in autofluorescence parameters associated with these fluorophores. We therefore extended our previous study to characterize the autofluorescence and diffuse reflectance signatures that accompany structural and functional remodeling of the heart in the initial four weeks following myocardial infarction – before collagen fluorescence overwhelms the NAD(P)H and FAD fluorescence signal. We highlight that the 16-week data set presented here (both myocardial infarction and age matched control groups) is the same as that reported in our earlier study [30] and is included here for comparison. The new results obtained at 1, 2 and 4 weeks post-infarction present the time evolution of the autofluorescence changes observed during the onset of heart failure. We believe that this more complete time-resolved characterization of this model should enhance the understanding of the spectroscopic changes that occur during remodeling post infarction.

## 2. Methods

All work involving animal use was carried out under protocols approved and regulated under the Animals (Scientific Procedures) Act 1986 (Project Licenses PPL 70/7399 and 70/7419).

### 2.1 Myocardial infarction induction surgery

Adult male Sprague-Dawley rats (250 – 300 g, Charles River UK, Ltd.) underwent proximal left anterior descending (LAD) coronary ligation to induce chronic myocardial infarction, as previously described [30,31]. This protocol was followed for all animals in this study, including the 16-week cohort. Anesthesia was induced with a mixture of 5% isoflurane and 95% oxygen in an induction chamber. The rat was then rapidly transferred to a Bain coaxial circuit and anesthesia was maintained with 2% isoflurane and 98% oxygen prior to intubation. Once anesthetized, 0.015‐0.030 mg buprenorphine (0.05 - 0.1 mg/kg), 1.25-1.5 mg enrofloxacin (5 mg/kg), and 2 ml of warmed 0.9% saline were administered subcutaneously. The rat was connected via the endotracheal cannula to a Harvard Ventilator (Harvard Apparatus, Massachusetts), and ventilated using volume-controlled ventilation with a minute volume of 180-250 mL/min (cycle rate 100/min; tidal volume 1.8 - 2.5 mL). A left parasternal incision was followed by division of the tissue planes, with retraction of pectoralis major and minor, to expose the intercostal muscles. A left thoracotomy was fashioned via the 4th intercostal space, and the pericardial sac divided. Anterior mobilization of the thymus gland was used to stabilize the heart and elevate the anterolateral aspect of the left ventricle into the center of the surgical field to facilitate the surgery. The LAD was ligated proximal to the first diagonal using a 7.0 Prolene suture at a point 1-2 mm distal to the left atrium inferior border along an axis parallel to the atrioventricular groove. Around 0.5-1.5 mm of myocardium was ensnared by the suture. Effective ligation-induced myocardial ischemia was confirmed by observing blanching and cyanosis of a significant sized ischemia zone in the LAD territory. If this was not observed, further ligations lateral and/or medial to the initial point are performed until a significant ischemia zone was induced. After successful coronary ligation, maintenance isoflurane was reduced to 1.0% during chest closure. Following surgery, rats were housed in single cages and in a stable environment with the standard facility 12/12 light-dark cycles, maintained on standard rat chow and water ad libitum, analgesia and checked daily for any signs of distress.

### 2.2 Preparatory surgery for in vivo measurements

The epicardial autofluorescence and white light diffuse reflectance characteristics of myocardial infarction animals (MI) and their age-matched controls (AMC) were studied in vivo during a terminal procedure at 1, 2, 4 and 16 weeks post-MI surgical induction. The number of animals studied at each time point is indicated in Table 1

**Table 1:** Number of animals studied for both experimental groups at each time point

Time post-MI	AMC	MI
1 week	3	3
2 weeks	4	3
4 weeks	4	3
16 weeks	6	6

. The animals were anesthetized by placement in an anesthetic chamber with 95%/5% O_2_/isoflurane. A blunt catheter was passed into the trachea under direct visualization and connected to a ventilator (Harvard Apparatus, USA). Anesthesia was maintained with 98%/2% O_2_/isoflurane using tidal volume of approximately 2 ml and a respiratory rate of approximately 90 cycles/min. Opiate analgesia was also given at appropriate dose for body weight. The heart and other internal organs were exposed via a laparotomy and sternotomy for spectroscopy measurements. At the end of each procedure the animal was sacrificed, and an apical section of the heart was taken for histology.

### 2.3 Optical instrumentation

The instrument used in this study consists of a single-point fiber-optic based time-resolved spectrofluorometer that was previously described elsewhere [30]. It includes two pulsed laser sources operated at 20 MHz and providing excitation light at 372 nm (LDH-P-C-375B, PicoQuant GmbH, Germany) and 438 nm (LDH-P-C-440B, PicoQuant GmbH, Germany). A bifurcated multimode fibre-optic bundle (NA = 0.22) consisting of three excitation fibers and fourteen collection fibers is used to deliver excitation light to the sample and to collect and deliver the resulting autofluorescence signal to the detection system. All optical fibers in the bundle have a core diameter of 200 μm. In the detection system, the autofluorescence signal is separated in three spectral bands that provide spectral resolution to the measurement: 410 ± 10 nm (channel 1), 455 ± 25 nm (channel 2) and 525 ± 25 nm (channel 3). At 438 nm excitation, only the fluorescence in the long emission wavelength band (525 ± 25 nm) is recorded, which is referred to as channel 4. Each detection channel consists of a cooled photon counting photomultiplier tube (PMT, PMC-100-1, Becker-Hickl GmbH, Germany). To provide temporal resolution to the fluorescence measurement, the electronic signals from each PMT are routed to a time-correlated single photon counting (TCSPC) acquisition card (SPC-830, Becker-Hickl GmbH, Germany). The particular set of wavelength emission bands was designed to maximize fluorescence collection from endogenous fluorophores of interest, namely collagen (channel 1), NAD(P)H (channels 2 and 3) and FAD (channel 4).

For white light diffuse reflectance measurements, the set-up also includes a white light source (HL-2000, Ocean Optics, USA) and a spectrometer (USB-2000 + , Ocean Optics, USA). The fiber-optic bundle used for autofluorescence measurements includes two additional multimode optical fibers that are used to deliver white light to the sample and the corresponding reflected signal to the spectrometer.

### 2.4 Data acquisition

**Fig. 1:**
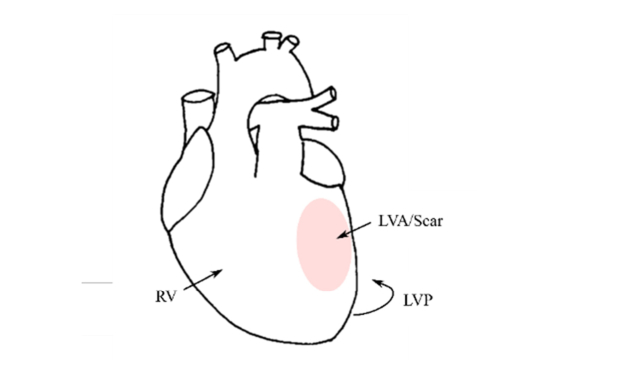
Diagram of the heart illustrating the regions of interest (ROI) studied. RV – Right ventricle; LVP – left ventricle posterior wall; LVA – left ventricle anterior wall. The region highlighted in red corresponds to the region of infarction.

Autofluorescence and white light diffuse reflectance measurements were recorded from three regions of interest (ROI): right ventricle (RV); left ventricle posterior wall (LVP), which is the remaining viable myocardium remote to the infarct scar; and the left ventricle anterior wall (LVA), which is the infarcted area containing the MI scar (Fig. 1

). All measurements were realized with the fiber optic probe in gentle contact with the epicardial surface of the heart. Autofluorescence and diffuse reflectance data were measured from three closely-placed random positions from each ROI and three repeat measurements were acquired at each position, resulting in a total of 9 sets of data for each ROI. For each ROI, the 9 diffuse reflectance spectra and fluorescence decays were analyzed independently and the results were averaged. The integration times used in a single acquisition were 1 second for 372 nm excitation, 1 second for 438 nm excitation and 50 milliseconds for white light diffuse reflectance measurements.

### 2.5 Data analysis

#### 2.5.1 Autofluorescence spectra

The relative autofluorescence intensity *F* in spectral channels 1, 2 and 3 was calculated as a fraction of the total autofluorescence signal detected for 372 nm excitation, as shown in Eq. (1):

$$F_{n}=\frac{I_{n}}{I_{1}+I_{2}+I_{3}}.$$ (1)

where *n* corresponds to the channel number (n = 1, 2, 3). We excluded channel 4 from ratio calculations as this is sensitive to any small day-to-day changes in the fiber-optic coupling efficiency and laser power differences between the two lasers of the instrument (channels 1-3 use a common excitation source and so are not affected by these factors).

#### 2.5.2 Autofluorescence lifetime

Fluorescence lifetime data were analyzed with software developed in-house using a non-linear least squares fitting routine from MATLAB (lsqnonlin function, R2011b, The Mathworks, Inc., USA) to minimize the goodness of fit *χ*^2^.

The fluorescence signal from biological tissue typically emanates from multiple fluorophore species, which results in a complex decay profile with multiple lifetime components, that can be described a multi-exponential decay model, as shown in Eq. (2):

I(t)=∑i=1nαie−tτi+C (2)

where *τ*_i_ and *α*_i_ refer to the fluorescence lifetime and pre-exponential factor for each component of the decay, respectively, and *C* is the measured background offset. Due to the relatively low number of photons collected in each spectral channel, particularly in channel 1, we chose to fit a single exponential decay model to channel 1 data and a double exponential decay model to the remaining channels. The model also included the measured instrument response function (IRF), afterpulsing probability of the detectors and incomplete decay estimation [30]. The intensity weighted mean lifetime *τ*_m_ (channels 2-4) was calculated as shown in Eq. (3):

$$\tau_{mean}=\frac{\alpha_{1}\tau_{1}^{2}+\alpha_{2}\tau_{2}^{2}}{\alpha_{1}\tau_{1}+\alpha_{2}\tau_{2}}$$ (3)

*τ*_1_, *τ*_2_ and *α*_1_, *α*_2_ refer to the fluorescence lifetimes and pre-exponential factors of the fast and slow components of the decay, respectively. The IRF for each detection channel was measured using DAPI (50 µM in water, Sigma-Aldrich, USA) for 372 nm excitation in all detection channels and Erythrosin B (50 µM in water, Sigma-Aldrich, USA) using 438 nm excitation. These reference dyes have short lifetimes (~200 ps), which are comparable to the pulse lengths of the lasers.

#### 2.5.3 White light diffuse reflectance

Diffuse reflectance spectra acquired from cardiac tissue, *I*(*λ*), were calibrated against the spectral response of the white light source, *I*_0_(*λ*), which was measured using a white reference target (WS-1-SL, Labsphere, USA) for each day of experiments. Tissue absorbance, *A*(*λ*), was calculated as shown in Eq. (4):

$$A\left(\lambda \right)=-\log_{10}\left(\frac{I\left(\lambda \right)}{I_{0}\left(\lambda \right)}\right)$$ (4)

As described in section 2.4, for each animal and ROI, three sets of diffuse reflectance measurements were acquired from each of three closely-placed random positions. The corresponding average spectrum from n = 9 measurements was taken to calculate the mean and standard deviation curves in Figs. 7–9. Data in Figs. 7–9 show mean and standard deviation over the number of animals reported in Table 1.

### 2.6 Statistical analysis

Statistical analysis was computed between AMC and MI hearts at each time point and region of interest with Welch’s t-test corrected for multiple comparisons using the Holm-Sidak test. The significance level was set to 0.05. Differences between groups were quantified using Cohen’s d effect size. Adjusted p-values and Cohen’s *d* coefficient for each comparison are presented in supplementary materials (see Data Availability section). The following notation was used to identify statistical significance between two sets of data: * p < 0.05; ** p < 0.01; *** p < 0.001; **** p < 0.0001.

### 2.7 Histology

Cardiac tissues were washed in sterile PBS, fixed in 10% paraformaldehyde for 48 hours and embedded in paraffin for sectioning. 6 µm thick sections were stained with picrosirius red (PSR) to demonstrate interstitial collagen content and imaged using a 10 × objective on a Zeiss Axio Observer inverted microscope with unpolarised illumination. Images from weeks 1-4 and 16 were acquired with different illumination settings. To facilitate visualization of key features in images from weeks 1-4, contrast was enhanced in post-processing using ImageJ [32].

## 3. Results

Occlusion of the left anterior descending (LAD) artery produced a myocardial infarction with formation of scar tissue in the anterior wall of the left ventricle in all MI specimens studied. Hypertrophy of the remaining myocardium was confirmed by increase in heart-to-body weight ratio in MI compared to AMC hearts, as indicated in Table 2

**Table 2:** Heart to body weight ratio demonstrate hypertrophy of the myocardium in MI specimens. All values are presented as mean ± standard deviation, in units of g/kg. NS – not significant.

Time post-MI	AMC	MI	p-value
1 week	3.36 ± 0.17	3.75 ± 0.22	NS
2 weeks	3.11 ± 0.47	4.07 ± 0.80	NS
4 weeks	2.33 ± 0.12	3.89 ± 0.38	0.002
16 weeks	2.84 ± 0.45	3.40 ± 0.60	NS

.

**Fig. 2:**
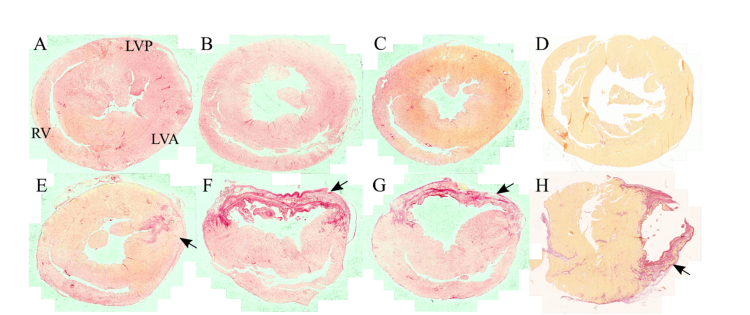
Representative histology images of AMC (A - D) and MI hearts (E - H) stained with PSR at weeks 1 (A, E), 2 (B, F), 4 (C, G) and 16 (D, H). Arrows on the bottom row indicate region of infarction (LVA) where scar tissue was formed, and deposition of collagen is more pronounced.

At week 1 post-MI induction, scar tissue in the LVA was only subtly visualized macroscopically. At week 2, a well-defined scar of white coloration started to form in the anterior wall of the left ventricle, thus becoming easier to identify macroscopically. In some cases, scar tissue was also visible in posterior wall of the left ventricle, suggesting development of the MI also in this region. At week 4, the scar tissue was clearly visible macroscopically but the edge of the scar (border zone) in the interventricular septum remained relatively diffuse. Finally, at 16 weeks post-MI induction surgery, a well-defined scar with clear demarcated edges was visible in all specimens in the LVA. Increase in interstitial collagen content was confirmed with histology in all MI specimens. Figure 2

shows representative histology images of whole heart sections stained with PSR to highlight collagen content. While in AMC hearts, collagen content is uniformly distributed, in MI hearts collagen is primarily found in the region of infarction.

### 3.1 Autofluorescence lifetime

#### 3.1.1 Right ventricle

Figure 3

**Fig. 3:**
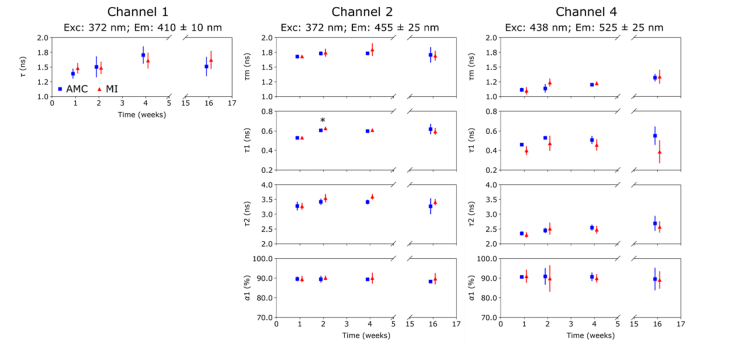
Autofluorescence lifetime parameters (see section on Data Analysis) measured in RV at 1, 2, 4 and 16 weeks.

shows the autofluorescence lifetime signature of the RV as a function of time following MI induction surgery for channels 1, 2 and 4. We have not included the results from channel 3 as this channel collects fluorescence from collagen, NAD(P)H and FAD, and so is not possible to separate their relative contributions or decay kinetics [24]. In general, the autofluorescence lifetime parameters appear to follow similar trends with time in AMC and MI hearts and any differences between the groups are subtle. In particular, we note a slight increase in lifetime of the long decay component *τ*_2_ of channel 2 in MI hearts, although not statistically significant. In contrast, the short component *τ*_1_ of channel 4 shows a lower fluorescence lifetime at all time points in MI hearts compared to AMC hearts.

#### 3.1.2 Left ventricle posterior wall (viable LV myocardium)

Figure 4

**Fig. 4:**
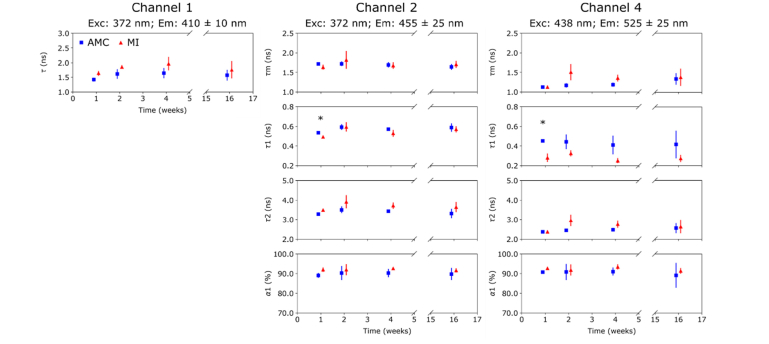
Autofluorescence lifetime parameters measured in LVP at 1, 2, 4 and 16 weeks. Differences in AMC and MI hearts are more pronounced compared to those observed for RV in all channels. In channel 4, we observe a general decrease in *τ*_1_ (all time points) and an increase in *τ*_2_ (weeks 2, 4 & 16) in MI relative to AMC hearts.

summarizes the autofluorescence lifetime signature measured in the LVP at each time point. Here, differences between AMC and MI hearts are more pronounced compared to the RV. A non-statistically significant increase in mean fluorescence lifetime in MI is observed in channel 1 at all time points relative to AMC hearts. A similar trend is seen in the long component *τ*_2_ of channel 2. Both could be attributed to increased collagen content due to the long fluorescence lifetime of collagen and its broad emission spectrum [33].

In channel 4, a shorter lifetime for the short decay component is seen at all time points in MI compared to AMC and this is statistically significant at week 1. This observation cannot be explained by increased collagen content and, given that the channel 4 signal is dominated by FAD autofluorescence, could be associated with metabolic alterations in the tissue. A non-statistically significant increase in the long decay component is seen in weeks 2 and 4, which could be attributed to increased collagen content. At week 16, the mean fluorescence lifetime is unchanged due to the decrease in *τ*_1_ being balanced by a slight decrease in its contribution.

#### 3.1.3 Left ventricle anterior wall (infarct scar)

**Fig. 5:**
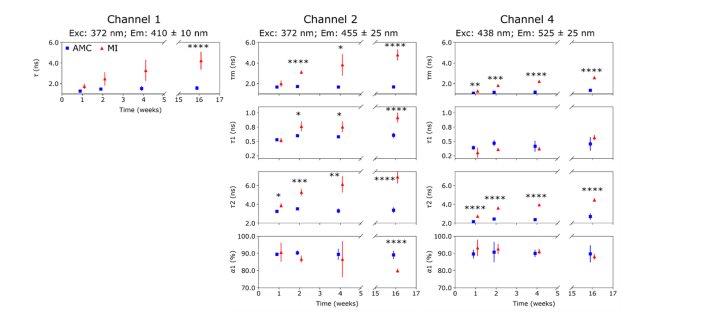
Autofluorescence lifetime parameters measured in LVA at 1, 2, 4 and 16 weeks. We observe a gradual increase in autofluorescence lifetime of MI hearts from week 1 to week 16, which suggests that collagen proliferation in the scar region starts immediately after MI induction. As in other regions, the lifetime component *τ*_1_ of channel 4 is shorter in MI than in AMC hearts at earlier time points. At week 16, extensive collagen proliferation leads to an increase in the fluorescence lifetime that possibly masks alterations in the fluorescence lifetime parameters caused by metabolic changes.

Differences between AMC and MI hearts are most clear in the LVA region of infarction, as shown in Fig. 5

. We observe a statistically significant increase of the mean autofluorescence lifetime in all detection channels of MI relative to AMC hearts. While differences in lifetime parameters are more pronounced at 16 weeks, the rate at which the changes occur is higher at earlier time points. At week 4, we measured mean autofluorescence lifetimes of 3.3 ± 1.0 ns, 3.8 ± 1.0 ns and 2.2 ± 0.1 ns for channels 1, 2 and 4, respectively, which correspond to 62%, 66% and 71% of the change in mean autofluorescence lifetimes between MI at week 1 and MI at 16 weeks (*τ_m_*_CH1_ = 4.2 ± 0.9 ns, *τ_m_*_CH2_ = 4.8 ± 0.5 ns, *τ_m_*_CH4_ = 2.6 ± 0.1 ns). In channel 2 we observe increasing lifetimes of the short and long decay components in MI hearts with time, accompanied by a decrease in the population of the short component. In channel 4, the lifetime component *τ*_1_ is shorter in MI relative to AMC hearts at 1 and 2 weeks and longer at week 16, which potentially reflects an initial decrease due to metabolic changes followed by an increase in collagen over time post-MI.

### 3.2 Autofluorescence spectra

**Fig. 6:**
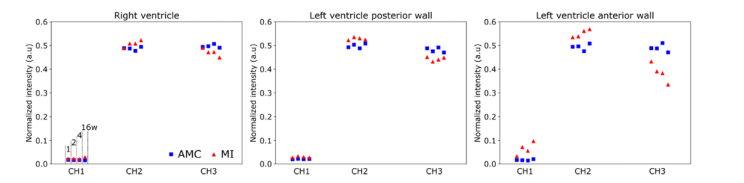
Normalized autofluorescence intensities in the RV, LVP and LVA under 372 nm excitation. Standard deviation bars are not included to avoid overcrowding the plots. Markers in each channel identify time point studied, from left (week 1) to right (week 16), as indicated in channel 1 of the right ventricle plot (left panel).

The autofluorescence intensity measured in channel 1-3 are presented normalized to the total autofluorescence signal detected using 372 nm excitation (see Eq. (1) as a function of time post MI, see Fig. 6

. Overall, we observe a blue shift in the emission spectra of MI relative to AMC hearts, with increased contribution from channel 2 and corresponding decrease in relative autofluorescence in channel 3. As with autofluorescence lifetime parameters, differences between AMC and MI hearts are more pronounced in the LVA, i.e. the region of infarction (Fig. 6, right plot), which suggests larger collagen deposition in this region as result of scarring. In the LVA there is an increase in the contribution of channel 1 to the autofluorescence signal. As expected, we observe a progressive blue shift in the autofluorescence spectra with time post-MI, which is more pronounced in the region of infarction but is also visible in RV. With respect to AMC hearts, it is interesting to observe the consistency of the normalized intensity values with region and ageing.

### 3.3 White light diffuse reflectance

#### 3.3.1 Right ventricle

**Fig. 7:**
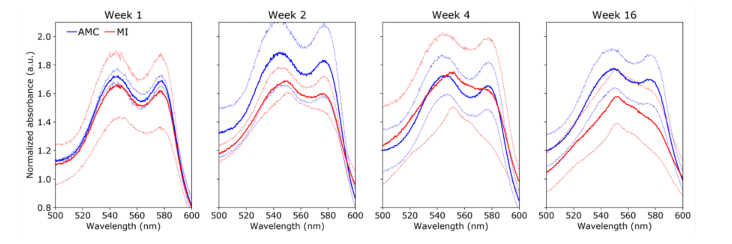
Normalized absorbance spectra in the RV between 500 and 600 nm for AMC (blue) and MI (red) hearts for different time points (left to right). Solid lines show average and dimmed lines show corresponding standard deviation curves.

In the RV, the absorbance spectra of AMC hearts (Fig. 7

) exhibit a relatively constant profile over time with relative maxima at approximately ~545 nm and ~578 nm, which are consistent with the spectral profiles of oxygenated hemoglobin and myoglobin [34]. In contrast, the absorbance spectra of MI hearts progressively change from a double peak profile at week 1 to a single peak at ~550 nm and a smooth shoulder at ~580 nm, at week 16. This complex spectral profile suggests contribution from multiple tissue absorbers. While the 580 nm shoulder could be associated with the presence oxyhemoglobin and oxymyoglobin, the peak at 550 nm peak is consistent with reduced cytochrome c absorption [35]. Cytochrome c is a key element of the electron transport chain and thus can provide a readout of the metabolic activity. Our results suggest an increased contribution of cytochrome c absorption with time post-MI, which may indicate a shift in metabolism in the remote RV.

##### Left ventricle posterior wall (viable LV myocardium)

**Fig. 8:**
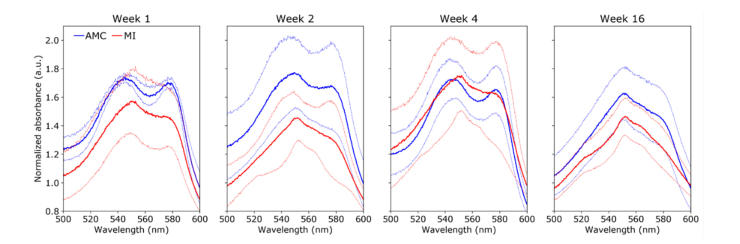
Normalized absorbance spectra in the LVP between 500 and 600 nm for AMC (blue) and MI (red) hearts for different time points (left to right). Solid lines show average and dimmed lines show corresponding standard deviation curves.

The absorbance spectra of MI hearts measured in the LVP (Fig. 8

) also indicate a progressive shift from a double peak profile at week 1 to a single peak at week 16. The increased contribution of cytochrome c is further emphasized by the presence of a smooth shoulder at ~520 nm, which is most evident in the lower standard deviation curves at weeks 2, 4 and 16, and is consistent with a well-defined peak in cytochrome c absorbance spectrum [35]. We note for both AMC and MI from week 2 onwards that the 580 nm peak/shoulder is less evident compared to the RV, which suggests a lower oxyhemoglobin/oxymyoglobin absorption resulting of a decrease in tissue oxygenation in this region with increasing age.

#### 3.3.2 Left ventricle anterior wall (infarct scar)

**Fig. 9:**
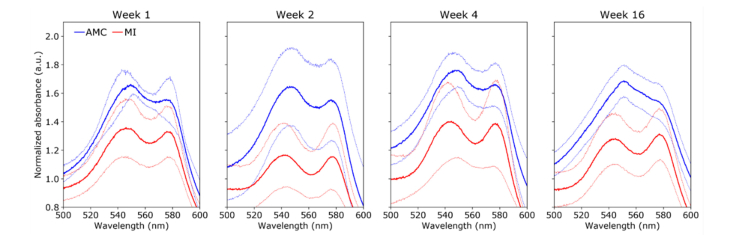
Normalized absorbance spectra in LVP between 500 and 600 nm for AMC (blue) and MI (red) hearts for different time points (left to right). Solid lines show average and dimmed lines show corresponding standard deviation curves.

The absorption spectra of MI hearts in the infarcted region (LVA) is different from those measured in remote non-infarcted regions (RV and LVP), as demonstrated in Fig. 9

. In particular, the absorbance spectra of MI hearts show hallmark oxyhemoglobin and oxymyoglobin absorption features at ~540 and ~580 nm, for all time points studied. Moreover, the prominent cytochrome c peak (~550 nm) that is visible at 16 weeks in both RV and LVP is not present in the mean absorbance spectra of the MI hearts. We also note a consistently lower absorbance of MI compared to AMC hearts, which indicates a lower concentration of absorbers owing to reduced blood content. In AMC hearts the first maximum peak is slightly red shifted compared to MI hearts, while the second peak remains at ~580 nm. These results suggest that while the tissue remains well-oxygenated, reduced cytochrome c has a considerable impact on the absorbance spectra of LVA in AMC hearts.

## 4. Discussion

In this investigation, we aimed to characterize the autofluorescence and diffuse reflectance signatures that accompany structural and functional remodeling of the heart following myocardial infarction. Our results demonstrate that autofluorescence lifetime has the sensitivity to detect structural and metabolic alterations in the infarcted area (LVA). In regions of viable myocardium remote to the infarct (RV and LVP), while results are not as striking as in infarcted areas, we observe trends in data that are suggestive of metabolic remodeling.

Structural alterations to the heart were confirmed with histology. Our histology data show a progressive thinning of the anterior wall of the left ventricle (black arrows in Fig. 2) accompanied by gradual increase in collagen content in this region, which is in agreement with previous observations [12]. The most striking differences occur from weeks 1 to 2. At week 1, all MI hearts showed evidence of collagen deposition that is localized to the region in the immediate vicinity of the lesion (Fig. 2(E)). Collagen deposition becomes more evident at week 2 (Fig. 2(F)), with sparse distribution of the collagen fibers through the entire thickness of the left ventricular wall. As a result, we observe a loss of cellularity and consequent thinning of the wall, which becomes more evident at weeks 4 and 16. In the remote regions RV and LVP, we did not observe significant changes to the collagen content from weeks 1 to 4, as expected from previous investigations, and our data therefore reflect the natural time course of remodeling in the viable non-infarcted myocardium [12].

Our autofluorescence data are in general agreement with histology observations and with previous studies [20]. In the region of infarction in the LVA, we observe a progressive and significant increase of the mean autofluorescence lifetimes of all spectral channels from week 1 to week 16 (Fig. 5). Similar trends are also visible in the long lifetime component *τ*_2_ of channels 2 and 4. These results are consistent with increasing collagen content in this region, given its long fluorescence lifetime and broad emission spectrum [30,36–38]. Similarly, our autofluorescence intensity data (Fig. 6) show a gradual blue shift in the emission spectra in the region of infarction of MI hearts relative to AMC, which is also consistent with increased collagen content, given the characteristic emission spectra of the endogenous fluorophores contributing to cardiac tissue autofluorescence i.e. collagen, NAD(P)H and FAD [39,40].

In myocardial regions remote to the scar (RV and LVP), differences between AMC and MI hearts are visible in the autofluorescence intensity data (Fig. 6), where we measured a blue shift in the emission spectra of MI hearts for all time points. Changes in the metabolic state of biological tissues are often inferred using the autofluorescence intensity of NAD(P)H relative to that of FAD i.e. optical redox ratio e.g [41,42], which, in our instrument, would correspond to the autofluorescence intensities measured in channels 2 and 3. However, given the potential interference of collagen autofluorescence, it is not possible to decouple the autofluorescence signal of collagen from those of NAD(P)H and FAD, making it challenging to attribute the origin of changes in the autofluorescence spectral measurements to either structural or metabolic alterations of the tissue. While our histology data do not show significant structural alterations in these regions, the spectral shift observed could be interpreted as a slight increase of collagenous content, which would be in agreement with previous investigations [12]. However, we did not measure any significant increase in the autofluorescence lifetime in channel 1 of MI hearts compared to AMC (Figs. 3 and 4) – as is seen in the LVA (Fig. 5) – as would be expected from an increase in collagenous tissue, given the long autofluorescence lifetime of collagen. Together with histology data, this result could indicate that changes in the autofluorescence spectra could be also be associated with metabolic changes. In a previous study in a Langendorff preparation [43] we have demonstrated that changes in the energetic state of the heart favoring glycolysis can cause a spectral blue shift, as a result of increased NADH contribution to the autofluorescence signal. Thus, early changes in the autofluorescence spectrum of remote regions may result from both structural and metabolic remodeling.

Changes in the autofluorescence decay parameters associated with functional alterations in the heart are most visible at earlier time points after MI and in regions remote to the infarction (RV and LVP), when the absolute increase in collagen content is less pronounced and thus the autofluorescence signal is less affected by this fluorophore. We observe a consistent reduction of the short lifetime component *τ*_1_ of channel 4 of MI hearts, which are opposite to the increase in fluorescence lifetime caused by collagen proliferation. The autofluorescence signal from healthy cardiac tissue detected in channel 4 emanates predominantly from FAD and the short lifetime component has previously been attributed to its protein-bound form [44]. Therefore, changes in this parameter are suggestive of an altered energetic state, as we previously demonstrated in the Langerdorff preparation [43]. Differences are most evident in the LVP viable myocardium (Fig. 4), where we measured a statistically significant decrease in MI hearts at week 1 (p < 0.05) post-MI. Similar differences were also observed at weeks 2, 4 and 16, albeit not statistically significant. This result suggests alteration of metabolism in the remaining viable myocardium following MI, possibly as part of the remodeling process. Overall, we see a similar but less pronounced change in the short lifetime component *τ*_1_ of channel 4 in RV (Fig. 3), which suggests that metabolic alterations also occur in this region as a response to the infarction. Changes in the short lifetime component *τ*_1_ of channel 4 are less evident in LVA (Fig. 4), where collagen proliferation was most pronounced. Nevertheless, we did observe a non-statistically significant decrease in *τ*_1_ of channel 4 at weeks 1 to 4. There is a consistent increase of *τ*_1_ over time in MI hearts from 303 ± 89 ps at week 1 to 566 ± 57 ps at week 16, while *τ*_1_ remained approximately constant around 410 ps in AMC hearts. The increase in *τ*_1_ lifetime in MI hearts is likely caused by increase in collagen content, consistent with histology, which further suggests that changes in this parameter at earlier time points are not associated with collagen, but rather to changes in metabolism. At week 16, extensive collagen deposition masks metabolic changes in this parameter caused by energetic alterations, resulting in a longer lifetime in MI hearts compared to AMC.

Multiple chromophores can contribute to the diffuse reflectance spectra of cardiac tissue, namely hemoglobin, myoglobin and cytochrome c [16,20,45]. These chromophores are sensitive to tissue oxygenation and thus their reduced and oxidized forms present distinct spectral features. Accordingly, the diffuse reflectance spectrum of the heart typically reflects the presence of all these chromophores, resulting in a complex spectral profile that is challenging to interpret. Our diffuse reflectance data are consistent with previous observations of this model [20]. In general, we observed a decrease of absorption in MI hearts that is more pronounced in the region of infarction (Fig. 9) and which we attribute to reduced tissue blood content following ischemia and consequent decrease in hemoglobin and loss of cellularity leading to reduced myoglobin content. The absorbance spectra of AMC hearts present characteristic features of oxygenated hemoglobin/myoglobin, with two relative maxima at ~540 nm and ~580 nm (Figs. 7–9, blue curves). Interestingly, the absorbance spectra measured in the region of infarction in MI hearts (Fig. 9, red curves) also resemble the spectra of oxyhemoglobin/oxymyoglobin, in all time points investigated. MI is a result of a decreased blood supply to the heart tissue and a decrease in tissue oxygenation in the absorbance spectra would therefore be expected in this region. However, ischemia in the region of infarction results in extensive cell loss and subsequent thinning of the myocardium wall, ultimately resulting in a lower concentration of tissue absorbers. Therefore, it is possible that our diffuse reflectance measurements from the infarcted area were probing beyond the cardiac wall, eventually reaching the left ventricular cavity, which was filled with well-oxygenated blood. In contrast, in remote regions to the infarction, we observed a shift in the emission spectra from a double peak profile at week 1 to a single peak at ~550 nm at week 16, which is consistent with an increased reduced state of the tissue. The 550 nm peak is a characteristic feature of reduced cytochrome c absorbance spectrum [16]. Cytochrome c plays an important role in the regulation of the mitochondrial respiratory chain and therefore can provide readouts of the metabolic activity [45,46]. The absorbance spectra of MI hearts measured in RV and LVP (Figs. 7 and 8) show a consistent shift towards a single peak at ~550 nm thus indicating an increase in cytochrome c contribution to the spectra, which, in turn, suggest a shift in cardiac energetics. In some measurements (e.g., Fig. 9, weeks 2 and 4), the absorbance spectra of MI hearts also present a shoulder at 580 nm, which suggests that the oxygenated forms of hemoglobin and myoglobin are dominant over their deoxygenated forms. Altogether, these results suggest increased metabolic activity with time post-MI in regions that are remote to the infarction, particularly in LVP viable myocardium, where changes in the absorbance spectra are more evident compared to RV.

In summary, this study demonstrates that time-resolved autofluorescence combined with diffuse reflectance measurements may offer a label-free readout of cardiac tissue structural and metabolic characteristics following myocardial infarction. We believe that this approach has significant potential for in vivo clinical measurements. For example, a label-free tool that could map the metabolic state of the myocardium in situ and in real time during cardiac surgery could be of great clinical interest.

## 5. Conclusions and outlook

In this work we have characterized the in vivo autofluorescence and diffuse reflectance signatures that accompany structural, functional and metabolic alterations in cardiac tissue at different time points following myocardial infarction. Structural alterations in the heart resulting from collagen proliferation are observed from the earliest stages after MI induction as an increase in the mean autofluorescence lifetime in all spectral channels. The large increase in collagen content masks gross changes in the autofluorescence of NAD(P)H and FAD caused by altered metabolism. These changes are observed as a decrease in the short lifetime of the autofluorescence decays associated with FAD autofluorescence and are more prominent at earlier stages after MI. Similarly, our diffuse reflectance data also suggest metabolic remodeling in the viable remaining myocardium with increased cytochrome c contribution with time following MI.

A multimodal setup employing time-resolved autofluorescence spectrofluorometry and diffuse reflectance spectroscopy may offer a label-free readout of cardiac tissue structural and metabolic aberrations. We aim to translate this approach into clinical settings to realize the first-in-man study of time-resolved autofluorescence in open-chest procedures at the time of cardiac surgery.

## Data availability

Supplementary materials and raw data underlying this publication are available from https://doi.org/10.5281/zenodo.1476463.
